# Hantaan virus activates Src family kinase and induces endothelial cell hyperpermeability via the TLR4/TRAF6 pathway

**DOI:** 10.1099/jmm.0.001989

**Published:** 2025-06-02

**Authors:** Xiaoyan Wang, Huanjun Shen, Hong Du, Hong Jiang, Pingzhong Wang, Ying Zhang

**Affiliations:** 1 Department of Infectious Diseases, Tangdu Hospital，The Fourth Military University, Xi’an 710038, PR China

**Keywords:** endothelial cells, Hantaan virus, haemorrhagic fever with renal syndrome, Src family kinase, toll-like receptor 4, vascular endothelial cadherin (VE-cadherin)

## Abstract

**Introduction.** Hantaan virus (HTNV) predominantly infects human vascular endothelial cells (ECs) and causes increased vascular permeability, triggering haemorrhagic fever with renal syndrome, mainly in Asia. Previous studies have shown that endothelial permeability is regulated in part by the break of cell–cell adherens junctions (AJs). However, the intracellular mechanisms by which HTNV induces EC hyperpermeability via AJs remain unclear.

**Hypothesis.** We hypothesize that HTNV activates TLR4, and its downstream TRAF6 interacts with SFK, leading to the phosphorylation of adhesion junction-associated proteins and increased cell permeability.

**Aim.** The present study aimed to investigate the molecular mechanism by which Src family kinases (SFKs) modulate AJs and affect permeability.

**Methodology.** Real-time PCR (RT-PCR) and Western blot were used to assess TLR4, TRAF6 and SFK expression; Western blot was used to analyse the protein expression of AJs; small interfering RNAs (siRNAs) were used to inhibit gene expression in the human umbilical vein endothelial cells (HUVECs) and the distribution of vascular endothelial cadherin (VE-cadherin) was observed by immunofluorescence.

**Results.** HUVECs infected by HTNV displayed a lower permeability after a siRNA knockdown of TLR4 (si-TLR4). Moreover, HTNV increased the expression of TRAF6 and the phosphorylation of Src and AJs. After siRNA knockdown of TRAF6 (si-TRAF6), a decrease in the phosphorylation of Src and VE-cadherin was observed in HTNV-infected ECs compared to that in siRNA controls.

**Conclusion.** These data, for the first time, indicated that HTNV-induced upregulation of AJ phosphorylation is regulated by the TLR4/TRAF6/SFK signalling pathway.

## Data Availability

All data generated or analysed during this study are included in this published article.

## Introduction

Hantaviruses belong to the *Bunyaviridae* family and are single-stranded, negative-sense, tri-segmented RNA viruses. The viral genomes consist of large (L), medium (M) and small (S) segments encoding RNA-dependent RNA polymerase, envelope glycoproteins (Gn and Gc) and nucleocapsid protein (N), respectively [1,2]. Hantaviruses lead to two severe febrile diseases worldwide: haemorrhagic fever with renal syndrome (HFRS) in Eurasia due to Old World hantavirus and hantavirus cardiopulmonary syndrome in the Americas due to New World hantavirus [3,4]. The two common types of hantavirus causing HFRS in China, Hantaan virus (HTNV) and Seoul virus, are carried by *Apodemus agrarius* and *Rattus norvegicus*, respectively [5]. HTNV tends to produce the most severe form of the disease in China during 1950–2014, with a mortality rate of 2.89%. Hantavirus mainly infects human vascular endothelial cells (ECs) and increases the permeability of microvascular endothelium. It has been reported that ECs isolated from adult and foetal veins are extremely vulnerable to HTNV infection. The increased permeability of ECs infected by HTNV is the main cause of endothelial barrier dysfunction rather than direct cellular cytotoxicity. Most importantly, the mechanism underlying the increased permeability caused by HTNV infection, whether resulting from the direct effects of the virus or the innate immune response to the infection, has yet to be established.

A previous study established that the combination of HTNV infection and vascular endothelial growth factor (VEGF) treatment significantly increases the permeability of EC monolayers in a time-dependent manner *in vitro* [6]; however, the specific mechanism is unclear. Another study revealed that TLR4 was activated in the innate response to HTNV and enhanced the production of IFN-*β*, IL-6 and TNF-*α* [7]. TLR4 may play an important role in the pathogenesis of HTNV through a MyD88-independent pathway, in which activating TRAF6, the downstream molecules of TRIF and expression of pro-inflammatory cytokines. These upregulated pro-inflammatory cytokines can cause permeability of endothelium either directly or indirectly through EC activation, resulting in the development of EC gaps. In addition, endothelial barrier function is an essential and tightly regulated process to maintain the homeostasis of blood vessels. Interendothelial junctions are composed of protein complexes of adherens junctions (AJs), tight junctions and gap junctions. Among them, AJs predominantly play a key role in endothelial barrier function. Reportedly, AJs regulate vascular permeability, in which vascular endothelial cadherin (VE-cadherin) plays a critical role by interacting with *β*-catenin through its cytoplasmic tail [8]. Nonetheless, the mechanism of how TLR4 participates in the pathogenesis of HTNV and increases EC permeability through the damage of endothelial cell–cell junctions is yet to be elucidated.

Src family kinases (SFKs) belong to the family of non-receptor tyrosine kinases and are crucial in the regulation of microvascular barrier function and various endothelial responses to a diversity of inflammatory mediators [9]. SFKs include c-Src, Fyn, Yes and Lyn [10], which were expressed in human microvascular EC. The structures of SFKs contain N-terminal myristoylated region, a unique region to each family member, an Src homology (SH3) domain, an SH2 domain, a short linker region, a catalytic domain and the regulatory C-terminus, which is highly conserved [11]. SFK is maintained in an inactive state under normal conditions and can be phosphorylated by intra- and intermolecular interactions. The kinase domain contains a Tyr^416^ within its activation loop that is autophosphorylated upon activation. SFK phosphorylates numerous substrates, such as VE-cadherin, talin and vinculin, that regulate the actin cytoskeleton, cell adhesion and cell movement in normal cells. Ikezoe *et al*. [12] reported that CsA increases vascular permeability in association with phosphorylation of Src/VE-cadherin, activation of NF-κB and upregulates the expression of cytokines in EA.hy926 ECs. In this study, we used ECs as a model to address the mechanism of permeability caused by HTNV.

## Methods

### Cell culture and viral infection

Human umbilical vein endothelial cells (HUVECs) were obtained from the American Type Culture Collection (Manassas, VA, USA) and cultured in endothelial cell medium (ECM) with 5% foetal bovine serum and 1% penicillin-streptomycin at 37 °C in 5% CO_2_ environment. ECM (Cat. No. 1001) was purchased from ScienCell (CA, USA). HTNV strain 76–118 was preserved in our laboratory (Centre of Infectious Diseases of Tangdu Hospital, Xi’an, China), and all related experiments were performed in a biosafety level 2 facility. HTNV litres were determined as described previously [13]. Briefly, tenfold serial dilutions of HTNV were added to a monolayer of E6 (Vero) cells in 24-well tissue culture plates. After co-incubation for 8 days, the infectivity was detected by the indirect indirect immunofluorescence assay (IFA) technique. In this study, HUVEC monolayers were infected by HTNV at a m.o.i. of 1 for 48 h, and>70% cells were found to be infected with HTNV as determined by immunofluorescence.

### Transient transfection

Small interfering RNAs (siRNAs) were introduced into HUVEC by transient transfection with X-tremeGENE HP DNA Transfection Reagent (Roche, Switzerland). HUVEC cells were transfected at 60% confluency. siRNA targeting human TLR4 and TRAF6 (5′−3′) were synthesized by GenePharma (Shanghai, China). siRNAs, prediluted in 250 µl of Opti-MEM, were combined with X-tremeGENE siRNA transfection reagent diluted in 250 µl of Opti-MEM I medium as recommended by the manufacturer. The mixture was incubated for 20 mins at room temperature for complex formation and added dropwise to cells. The final siRNA concentration was 80 nM. At 48 h post-transfection, cells were collected and followed by Western blot analysis or real-time PCR (RT-PCR).

### Real-time PCR

Total RNA was extracted from cell line HUVEC using the Qiagen RNeasy kit following the manufacturer’s instructions. The quality of the extracted RNA was assessed on a ScanDrop (Analytik Jena, Germany). An equivalent of 200 ng of the total RNA was reverse-transcribed into cDNA using one-step RT-PCR kit (TaKaRa) with oligo dT primer following the manufacturer’s protocol. The 25 µl reaction consisted of 1 µl of the diluted cDNA product, 12.5 μl of 2×Power SYBR^®^ Green PCR Master Mix, 1 µl each of forward and reverse primers (10 µM) and 9.5 µl of nuclease-free water. Primer sequences are listed in Table 1. *GAPDH* was used as an internal control in all experiments. The amplification conditions were as follows: 95 °C for 30 s and 40 cycles of 95 °C for 5 s and 60 °C for 30 s. The fluorescence signal emitted was collected by the Bio-IQ5 Detection System and converted into numerical values.

**Table 1.** PCR primers used in this study

Genes	Primer sequences
*TLR4*	F: 5′-CAGTGCTTCCTGCTCTTT-3′
	R: 5′-GGTTTCTTCTCCCATCCT-3′
*TRAF6*	F: 5′-GATGCAGAGGAATCACTTGGC-3′
	R: 5′-GGTCTGTCTTACAAGGCGAC-3′
*SRC*	F: 5′-GTACTTTGTCCCGTGGCATTT-3′
	R: 5′-TTTTTTTCTGAGGTCTGTTTG-3′
*YES*	F: 5′-AACAAGTGGAGC GCGAGGATACA-3′
	R: 5′-TGGGGAAAAAAAGAAAGGAAA-3′
*LYN*	F: 5′-ATTTCATTCTGTGTGTTTTGT-3′
	R: 5′-GGAGTATGGATTCTGTCTTTC-3′
*FYN*	F: 5′-AAAGGACCCTGAAGAACGCCC-3′
	R: 5′-CAGTCTCCA CATTAGCACCCA-3′
*GAPDH*	F: 5′-CTTAGCACCCCTGGCCAAG-3′
	R: 5′-GATGTTCTGGAGAGCCCCG-3′

### Western blotting

After various treatments, HUVECs were lysed in radio-immunoprecipitation assay buffer (P0013B, Beyotime) for 10 mins, denatured and clarified by centrifugation at 12,000 r.p.m. for 10 mins. An equivalent of 20 µg protein/lane was separated by 10% SDS-PAGE (P0012A, Beyotime) and transferred onto 0.45 µm PVDF membrane (IPVH00010, Millipore; Bedford, MA, USA). After blocking with 5% BSA (non-fat milk) in PBS, the membranes were probed with optimal primary antibodies at 4 °C overnight and then incubated with horse radish peroxidase (HRP) conjugated anti-rabbit or anti-mouse IgG antibodies at room temperature for 2 h. The immunoreactive bands were developed by enhanced chemiluminescence reagents for 5 mins. The primary antibodies against TRAF6, Src, Yes, Fyn, Lyn, Phospho-Src Family, VE-cadherin, *β*-catenin and *γ*-catenin were purchased from Cell Signaling Technology (CST), and those against *β*-catenin (phospho Y142), *γ*-catenin (phospho Y550), p120-catenin, p120-catenin (phospho Y228), VE-cadherin (phospho Y658) and HRP-conjugated secondary Abs were purchased from Abcam (Cambridge, MA, USA). The primary antibodies were used at 1:1,000 dilution, while the secondary antibodies were used at 1:5,000 dilution. The intensities of the proteins detected on X-ray films were calculated by densitometric analysis using ImageJ (ImageJ 1.42q).

### Immunofluorescence and image analysis

After treatment with HTNV, HUVECs were fixed in 4% formaldehyde at room temperature for 15 mins and permeabilized with 0.3% Triton X-100 in PBS at room temperature for 5 mins. After blocking with 5% BSA in PBS, the cells were incubated with rabbit anti-VE-cadherin (diluted 1:100; CST) to visualize the distribution of the protein. After incubation with the primary antibody, the cells were incubated with FITC-conjugated goat anti-rabbit IgG (CST) in the dark for 1 h. Then, the coverslips were mounted in the medium using 4,6-diamino-2-phenylindole (DAPI, Thermo Fisher) to label cell nuclei. Finally, coverslips were observed and photographed under an OLYMPUS microscope (BX51+DP70, Olympus, Tokyo, Japan).

### EC permeability assay *in vitro*

To analyse the effect of HTNV activation on cellular permeability, HUVECs were seeded into the upper chamber of the transwell (polycarbonate filters, 0.4 mm pores; Millipore) at a density of 5×10^6^ cells ml^−1^ and grown in ECM (ScienCell™). The cells reached 80–90% confluency after 5–7 days and were then treated with siRNA. At the start of the experiment, the culture medium in the upper compartment was replaced with HTNV. After incubation, HRP conjugated to goat immunoglobulin (8 mg ml^−1^ initial concentration in the upper chamber; MW=200 kDa; specific activity 28 units ml^−1^) was added to the upper chamber. After incubation at 37 °C for 1 h, the medium in the lower compartment was assayed for the presence of HRP activity using O-phenylenediamine (OPD) as the chromogenic substrate.

### Statistical analysis

All values are expressed as the mean±standard of at least three independent biological replicates. Statistical analysis was carried out using GraphPad Prism^®^ 6.0 software (GraphPad Inc., CA, USA). One-way ANOVA or Student’s t-test was used to analyse the differences between groups. *P*<0.05 indicated a statistically significant difference.

## Results

### TLR4 knockdown and its effect on HTNV-induced EC permeability

**Fig. 1.**
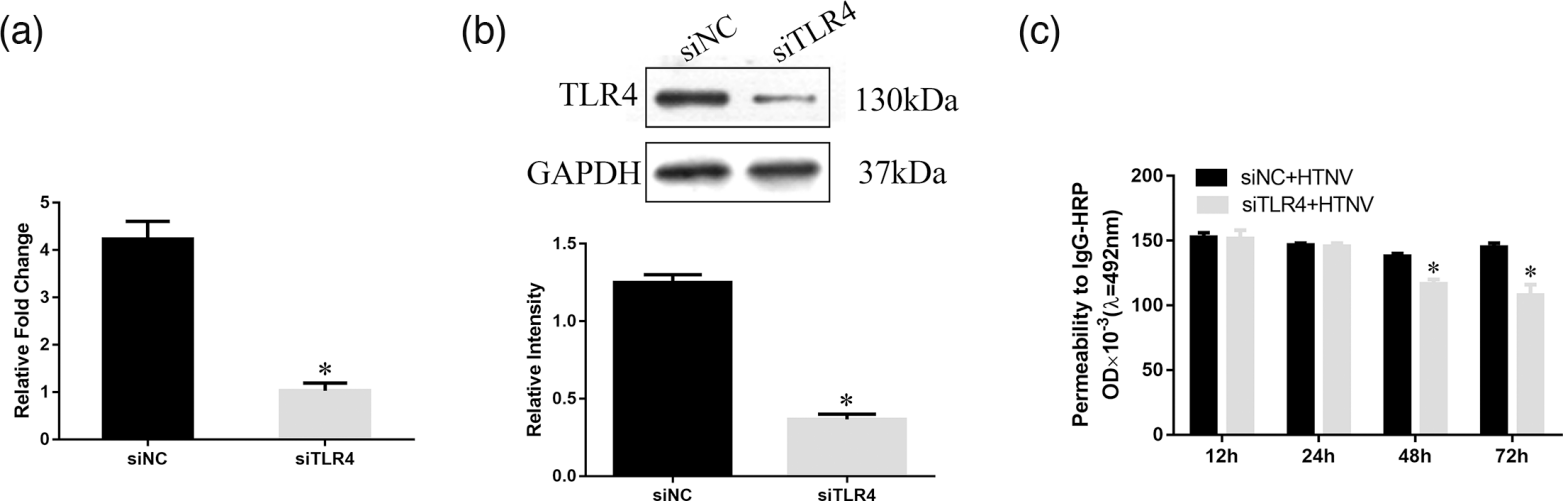
TLR4 knockdown and its effect on HTNV-induced EC permeability. (a) RT-PCR of TLR4 when HUVECs were transfected with TLR4 siRNAs or a corresponding negative control. (b) On the upper part, Western blot of TLR4 when HUVECs were transfected with TLR4 siRNAs or a corresponding negative control. The lower part is the quantification of TLR4. (c) Permeability of HUVECs measured by IgG-HRP. * indicates *P*<0.05 compared to controls.

In a previous study, we reported that HTNV exerted time-dependent effects on the monolayer permeability of ECs [14]. Herein, we determined if knocking down TLR4 blocks HTNV-induced EC permeability. First, we transfected ECs with control or TLR4-specific siRNAs, and then EC permeability induced by HTNV was assayed by HRP trans-endothelial flux. Quantitative RT-PCR analysis indicated that ECs transfected with TLR4 siRNAs specifically reduced *TLR4* mRNA levels by 75% (Fig. 1a). Fig. 1(b) shows that TLR4 siRNA has a significant effect on the expression of TLR4 protein. In addition, we found that TLR4 siRNA reduced the hyperpermeability of HTNV-infected ECs at 48 h (Fig. 1c). These results suggested that TLR4 is related to the HTNV-induced permeability increase in ECs.

### HTNV infection upregulates TRAF6 in HUVEC via TLR4

**Fig. 2.**
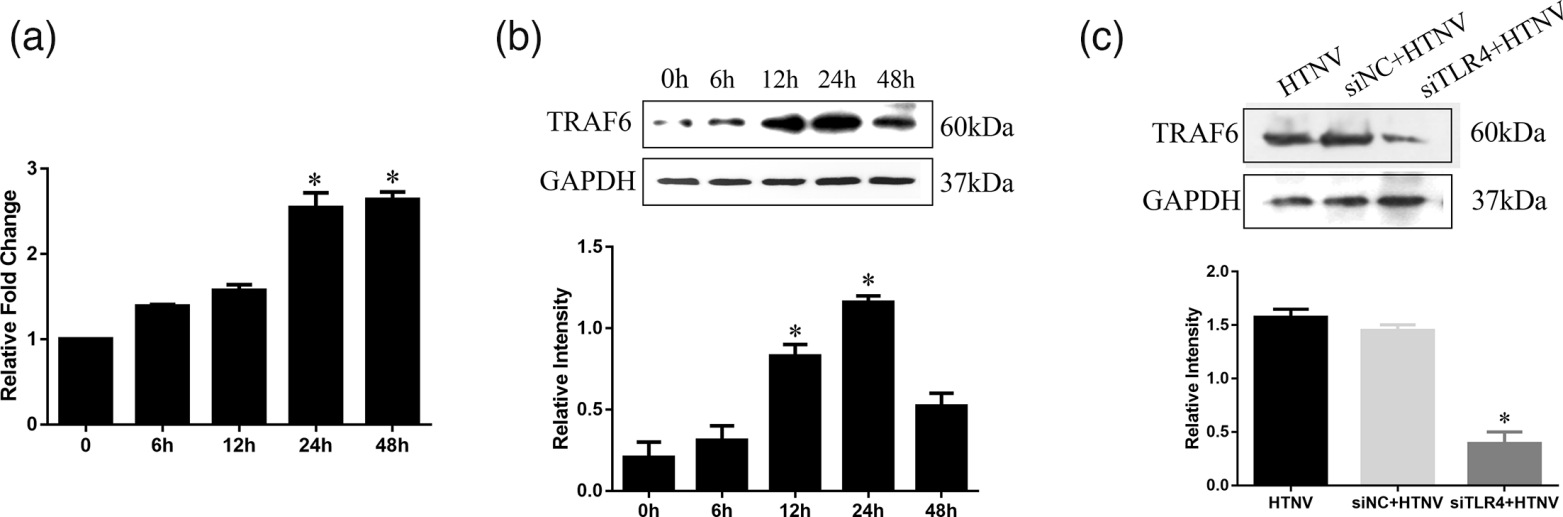
Effects of HTNV on TRAF6 in HUVECs. (a) RT-PCR and (b) Western blot were used to detect TRAF6 when HUVECs were incubated with HTNV for 6, 12, 24 and 48 h. (c) Western blotting was used to detect TRAF6 when HUVECs were transfected with si-TLR4. * indicates *P*<0.05.

Our previous study demonstrated that the *TLR4* mRNA expression was approximately threefold higher after HTNV infection than in uninfected cells [15]. Moreover, HTNV activated TLR4 signalling in a MyD88-independent manner [15]. To determine the role of TRAF6, the downstream protein in the MyD88-independent pathway, quantitative RT-PCR and Western blotting were used. Quantitative RT-PCR analysis demonstrated that *TRAF6* mRNA was increased at 6 h, peaked at 24 h and then maintained up to 48 h following HTNV infections (Fig. 2a). Fig. 2(b) shows the peak of TRAF6 expression at 24 h while reversing the increasing trend at 48 h. Taken together, TRAF6 increased gradually in a time-dependent manner and peaked at 24 h in ECs after HTNV infection. Furthermore, we found that knocking down TLR4 with the specific siRNA reduces the HTNV-induced TRAF6 expression compared to siRNA negative control (siNC) (Fig. 2c). These data indicated that HTNV activates the TLR4–TRAF6 signalling pathway.

### HTNV infection upregulates SFKs in HUVECs

**Fig. 3.**
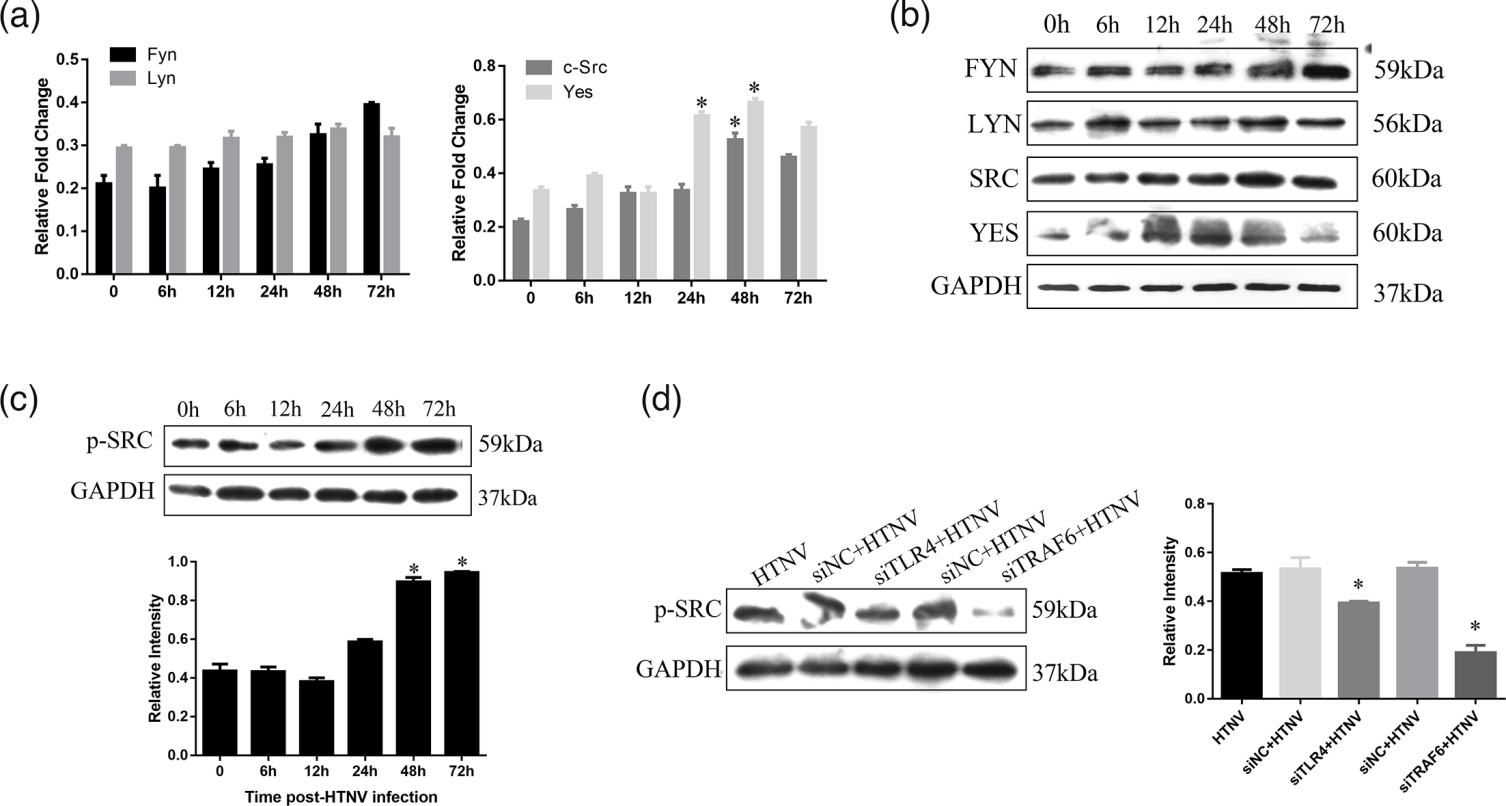
Influence of HTNV on the expression of SFK family in HUVECs. (a) RT-PCR and (b) Western blot were used to detect the SFK family (Yes, Src, Lyn and Fyn) when HUVECs were incubated with HTNV for 6, 12, 24, 48 and 72 h. Western blotting was used to detect SFK phosphorylation when HUVECs were (c) incubated with HTNV for different time points and (d) transfected with si-TRAF6 and si-TLR4. * indicates *P*<0.05.

SFK and its phosphorylation at Tyr^419^ regulated the permeability of vascular ECs. In order to assess the role of Src in HTNV-induced cell hyperpermeability, we examined the phosphorylation of SFK members after HTNV infection. HUVECs were incubated with HTNV for 6, 12, 24, 48 and 72 h and solubilized in lysis buffer for quantitative RT-PCR and Western blotting. The results of RT-PCR (Fig. 3a) showed that *Yes* and *Src* were upregulated significantly at 24 and 48 h, respectively, after HTNV infection, while Fyn and Lyn altered only slightly. As shown in Fig. 3(b), the protein and mRNA expression of SFK members was similar. Moreover, the phosphorylation of Src Tyr^419^ showed a gradually increasing trend that peaked at 48 h after HTNV infection (Fig. 3c). These findings indicated that SFK was activated in HTNV infection. Next, we asked whether TLR4 and TRAF6 affected SFK activation. HUVECs were transfected with TLR4 and TRAF6 siRNAs, and the phosphorylation of Src was detected using Western blotting. We found that knocking down TLR4 and TRAF6 reduced SFK phosphorylation at 48 h (Fig. 3d). These data suggested that Src activation may be via the TLR4–TRAF6 pathway in HTNV infection.

### HTNV modifies AJ protein

**Fig. 4.**
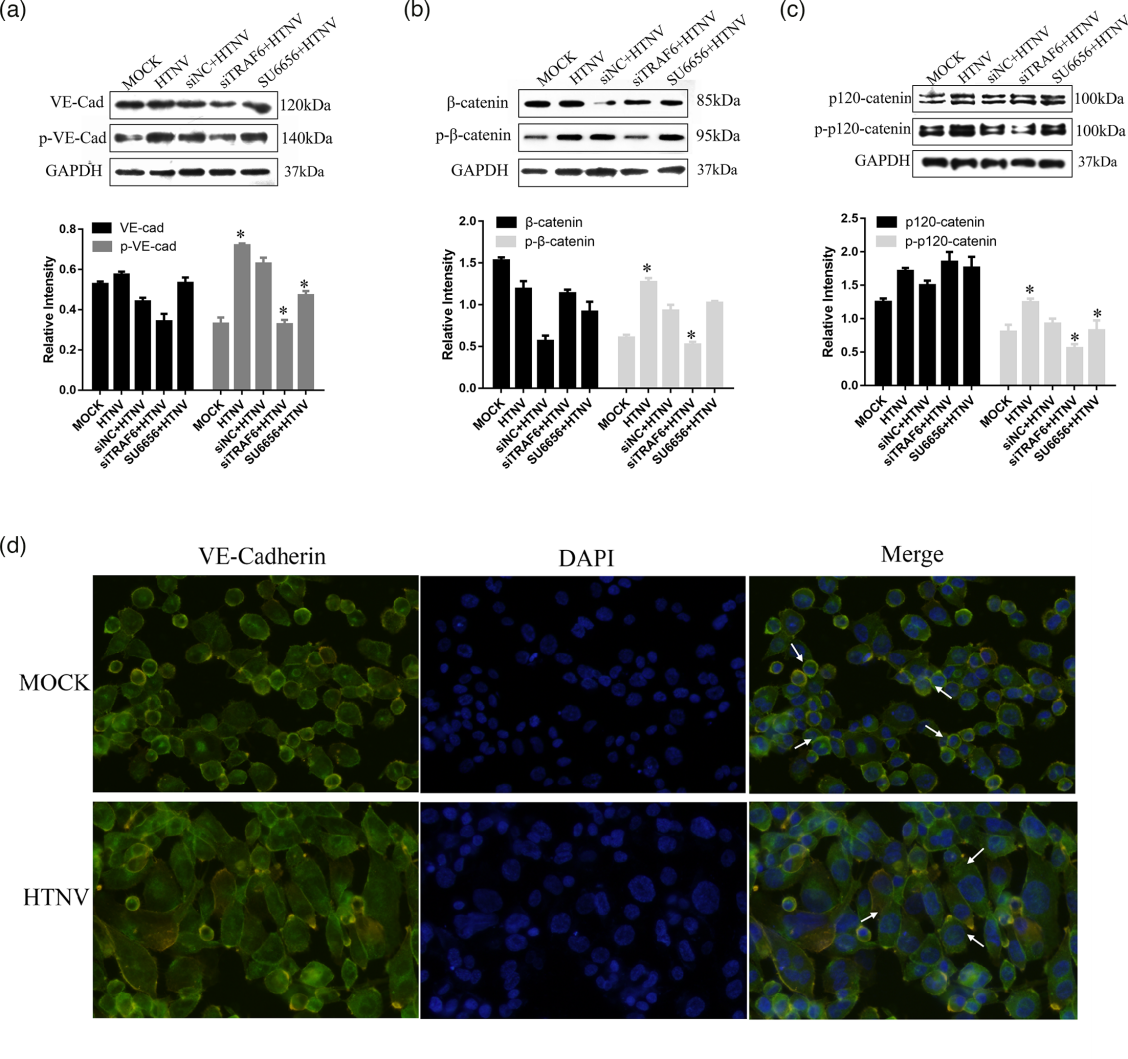
Expression of the AJ family after HUVECs were infected with HTNV. Western blotting was used to detect AJs, and their phosphorylation includes (a) VE-cadherin, (b) *β*-catenin and (c) p120-catenin when HUVECs were incubated with HTNV for 48 h or transfected with si-TRAF6 and siNC or added with SU6656. (d) Immunofluorescence of VE-cadherin after HUVECs infected by HTNV. Green indicates cells labelled with VE-cadherin; blue, DAPI; white arrow: distribution of VE-cadherin. * indicates *P*<0.05.

Given that Src acted upstream of VE-cadherin and caused the disruption of the endothelial barrier, we investigated whether Src influenced the distribution of VE-cadherin after HTNV infection. First, HUVECs were transfected with si-TRAF6 and its corresponding negative control to knock down TRAF6 or added SU6656 to block SFK before HTNV infection for 24 h. Then, Western blotting was used to detect the changes in AJ protein complex, including VE-cadherin and its adaptors, *β*-catenin and p120-catenin. Our data showed that HTNV increases the phosphorylation of VE-cadherin, *β*-catenin and p120-catenin, but TRAF6 knockdown (si-TRAF6) decreases VE-cadherin phosphorylation compared to mock-transfected cells (Fig. 4a–c). Additionally, pretreatment with SU6656 for 2 h significantly depressed the phosphorylation of AJ. These data suggested that TRAF6 and SFK participate in HTNV-induced AJ phosphorylation. In order to observe the changes in VE-cadherin visually, we detected the protein distribution using immunofluorescence (Fig. 4d). Under control conditions, VE-cadherin appears as a continuous line along the interendothelial cell border. However, treatment with HTNV for 48 h reduced and interrupted the distribution of VE-cadherin. These data suggested that redistribution of VE-cadherin mediates HTNV-induced endothelial hyperpermeability, and the tyrosine kinase activity of Src triggers the downstream signalling events.

## Discussion

HFRS caused by hantavirus infection causes a non-pathogenic, persistent infection in rodents. On the other hand, typical symptoms of HRFS in humans are fever and renal failure, including five progressive stages: febrile stage, hypotensive stage, oliguric stage, diuretic stage and convalescent stage. Some of these stages may overlap or two phases may be missing in clinical cases. Renal failure and haemorrhage are the hallmarks of HFRS disease and are caused by vascular hyperpermeability. However, the pathogenesis of HFRS has not yet been elucidated. HTNV mainly infects vascular ECs and causes damage. *In vitro*, ECs infected by HTNV dramatically enhance permeability in response to VEGF. Several reviews have summarized that endothelial permeability is closely related to the disassembly of AJs [16,17]. Whether the vascular endothelial permeability changes are caused by HTNV remains unknown. In this study, we found that HTNV activates TLR4, and its downstream TRAF6 interacts with SFK, leading to the phosphorylation of adhesion junction-associated proteins, including VE-cadherin, and increased cell permeability.

TLRs are a family of innate immunity receptors that can identify pathogen-associated molecular patterns and trigger signal transduction, thereby releasing inflammatory mediators, including cytokines, and producing a series of immune and pathological reactions [18]. A previous study [19] showed that LPS increases paracellular permeability through TLR4-mediated SFK activation in human lung microvascular endothelia, indicating that TLR4 participates in the barrier response. Another study demonstrated that TLR4 plays a role in the pathogenesis of HTNV infection [7]. The TLR4 knockdown resulted in lower permeability of HUVECs, which further validated the above observation. A previous study reported that HTNV induces TLR4-mediated activation of NF-κB and IRF-3 by a MyD88-independent signalling pathway [14]. The MyD88-independent signalling pathway includes TRIF, followed by two branches, TRAF6 and TRAF3. In classical TLR4 signalling, LPS recruits TRAF6 into the cytoplasm and activates NF-κB and the mitogen-activated protein kinase (MAPK) family [20]. TRAF6 acts as a key mediator of vascular endothelial pathophysiology [21,23]. Our combined data indicated that TRAF6 is gradually increased with HTNV infection in a time-dependent manner after ECs. Interestingly, TRAF6 expression is considerably decreased at 48 h. This could be either due to miR-146a-5p or miR-146b-5p silencing TRAF6 mRNA or TRAF6 self-ubiquitination mediating protein degradation through the proteasome pathway [24,25]. HUVECs transfected with TLR4 siRNA showed lower expression of TRAF6 and a slight decrease in EC permeability caused by HTNV. These findings indicated HTNV-induced hyperpermeability of EC monolayer via TLR4–TRAF6 pathway. Furthermore, TRAF6 and c-Src have been reported to interact physically in signalling pathways in non-EC systems [26,27].

Reportedly, SFK is an adaptor protein in the TLR4 signal transduction process, initiating the pro-inflammatory response [28]. Previous studies have shown that the activation of SFK contributes to pro-inflammatory increased EC permeability [29,30]. Although it is well accepted that endothelial SFK activation plays an important role in the regulation of vascular permeability, the mechanisms that ultimately lead to the loss of barrier function are still under investigation. In the present study, transcription of Src and Yes showed an increase after HTNV infection, whereas Lyn and Fyn remained unchanged. This phenomenon was consistent with the finding that Src and Yes promote a loss of barrier function in some models, while Lyn is required to sustain the endothelial barrier, and Fyn increases permeability [31,32]. Importantly, Src phosphorylation at Tyr^419^ was activated in a time-dependent manner in HUVECs, which was relevant to the phosphorylation of VE-cadherin. Our study also showed that transfection of TLR4 and TRAF6 siRNAs partially abated HTNV-induced Src phosphorylation in HUVECs. This finding is consistent with the observation that prior knockdown of TRAF6 blocked LPS-induced SFK activation compared to the control siRNA-transfected cells [19]. These data demonstrated that the TLR4–TRAF6 pathway is required for HTNV-induced Src phosphorylation.

The barrier function of the endothelial monolayer is regulated by cell–cell and cell-extracellular matrix adhesion. The AJs joining the neighbouring cells are mainly formed by VE-cadherin, linked through its cytoplasmic tail to the p120 and *β*-catenin. Furthermore, the tyrosine phosphorylation of VE-cadherin and other components of AJs was associated with weak junctions and impaired barrier function. A previous study described that VE-cadherin was gradually downregulated at the mRNA and protein levels in HTNV-infected cells following VEGF stimulation [33]. Also, VE-cadherin was phosphorylated by Src on tyrosine 685 when activated by VEGF in ECs [34]. In the present study, the expression of VE-cadherin and p120-catenin showed a slight increase, whereas the phosphorylation of AJs proteins was increased after HTNV infection; this phenomenon was related to increased endothelial permeability. In order to examine if TRAF6 and SFK activity had a role in HTNV-increased permeability, we used TRAF6 siRNA and the SFK inhibitor SU6656 to detect the phosphorylation of AJs. The pretreatment with TRAF6 siRNA or SU6656 suppressed HTNV-induced VE-cadherin phosphorylation, indicating decreased permeability. These results demonstrated a signalling mechanism of HTNV-induced Src-dependent VE-cadherin phosphorylation. Hence, SFK activation should be considered a potential driver for altering EC barrier function after HTNV infection.

Taken together, the HTNV-inducible TLR4–TRAF6 pathway activates Src kinase and participates in increased tyrosine phosphorylation of AJ proteins, leading to the opening of the paracellular pathway in human vascular ECs. This study identifies a new potential mechanism of vascular leakage, which contributes to understanding the prevention and therapy of HTNV-associated haemorrhage. Nonetheless, the study is based on a cell model, so it is limited in its conclusions for infection within a human. Therefore, additional studies are required to evaluate whether the SFK inhibitor contributes to permeability in the animal model.

## Abbreviations

AJs: adherens junctions
CST: Cell Signaling Technology
DAPI: 4,6-diamino-2-phenylindole
ECM: endothelial cell medium
ECs: endothelial cells
HFRS: haemorrhagic fever with renal syndrome
HRP: horse radish peroxidase
HTNV: Hantaan virus
HUVECs: human umbilical vein endothelial cells
IFA: indirect immunofluorescence assay
MAPK: mitogen-activated protein kinase
OPD: O-phenylenediamine
RT-PCR: real-time PCR
SFKs: Src family kinases
siRNAs: small interfering RNAs
VE-cadherin: vascular endothelial cadherin
VEGF: vascular endothelial growth factor
